# Gingival margin stabilization using the final prosthetic restoration (BOPT). A case report

**DOI:** 10.4317/jced.61837

**Published:** 2024-08-01

**Authors:** María Granell-Ruiz, Ruggero Bertolini, Cristina Rech-Ortega, Begoña Oteiza-Galdón, Kheira Bouazza-Juanes

**Affiliations:** 1 Universidad Europea de Valencia. Faculty of Health Sciences. Department of Dentistry; 2 Clinical and Applied in Dental and Implant-Prosthetics Research Group. Universidad Europea de Valencia. Faculty of Health Sciences. Department of Dentistry

## Abstract

One of the most contentious and extensively discussed topics in the field of dentistry when fabricating prosthetic restorations is the location and design of the finishing line in relation to the gingival tissues. Upon completion of the temporary crown and subsequent fabrication of the final restoration, two potential issues may arise: 1) the analog or digital impression may not accurately reflect the shape of the gingiva obtained with the temporary crown due to gingival collapse upon crown removal, even in the presence of retraction cords; and 2) the desired gingival shape may not have been achieved with the temporary crown. The objective of this article is to describe the stabilization of gingival tissues following twelve weeks of clinical observation. During this period, the provisional crown is recontoured twice in the apical-coronal direction with a four-week interval. This approach allows for the growth of sufficient gingival tissue in the horizontal direction at the point of the vestibular emergence profile, which will then stabilize once more following a slight recontouring of the final restoration, which will be performed in the clinic. The amount of gingival adaptation is not quantifiable in a numerical sense; rather, it is directly proportional to the amount of tissue that can be obtained with the new emergence profile of the temporary crown. The outcome is contingent upon the operator and there is no fixed quantity that can be achieved in every instance. In essence, there is no fixed numerical value that can be relied upon to lower the gingival parabola in the apical-coronal direction through the adaptation of tissues to the new shape of the temporary crown emergence profile.

** Key words:**Vertical preparation, BOPT technique, tissue stabilization, final restoration.

## Introduction

One of the most contentious and extensively discussed topics in the field of dentistry when fabricating prosthetic restorations is the location and design of the finishing line in relation to the gingival tissues (1).

Two principal trends have emerged in dental preparations throughout history. The first is characterized by a clearly defined finishing line, while the second is defined by a vertical preparation without a visible line but with a finishing area (2). Among the vertical preparations, two distinct types are described in the literature, with their definitions provided in the glossary of prosthodontic terms (1):

• FEATHER-EDGE FINISH LINE: the demarcation between prepared and unprepared tooth structure created by minimal tooth preparation without a defined visible line of reference for the cavosurface finish line such as a shoulder or chamfer finish line.

• KNIFE-EDGE FINISH LINE: a clearly defined junction of prepared and unprepared tooth structure that lacks a concavity at the gingival termination.

According to Shillinburg, vertical preparations have always been considered inappropriate for the fabrication of metal-ceramic or all-ceramic crowns. This is due to the lack of marginal adaptation, horizontal overcontouring, and the potential distortion of the ceramic during firing, which can negatively impact periodontal health (3). However, recent studies have demonstrated that restorations with vertical preparations offer superior periodontal health compared to those with horizontal preparations (4).

Historical introduction of vertical preparations

One of the first published articles to discuss vertical preparations was by Morton Amsterdam and Luis Abrams, members of the working group of M. Goldman and W. Cohen of the Department of Periodontology at the University of Pennsylvania. Their article described the possibility of performing a vertical preparation on periodontal teeth without periodontal surgery (5).

Subsequently, in 1974, V. Pollard conducted a study to identify the optimal characteristics of the diamond burs to perform a correct gingival curettage of the epithelial sulcus. This procedure was performed simultaneously with tooth preparation.

In the 1980s, the technique was revived by Rex Ingraham. In his article, Ingraham described a gingival curettage procedure with rotary instruments. This technique was designed to prepare the tooth and the gingival sulcus. In practice, the procedure was performed in the intraepithelial sulcus using a bur to create an “elongated chamfer” (6).

In the 1990s, the working group of the Porta Mascarella School of Bologna in Italy, with Di Febo and Carnevale, adopted a perio-prosthetic clinical protocol for cases with severe periodontal compromise. The protocol called for the preparation of teeth at the level of the osseous crest after the creation of a flap. This was done to eliminate undercuts, facilitate the final preparation, and the impression. The protocol involved deepening the “barrelling-in” (accentuated preparation of the existing anatomical concavities on the coronal body at the bifurcation), correcting the root proximities of adjacent teeth, and reducing the concavity of the roots. Subsequently, the tissues were allowed to heal. If possible, a slight chamfer was made on the tooth as a finishing line for the prosthetic margin 8-12 weeks after surgery. In contrast, if the residual tooth structure was insufficient to permit the chamfer to be made, the vertical preparation made during surgery also served as the final prosthetic preparation (7,8).

In 2013, Ignazio Loi introduced his personal philosophy of vertical preparation, which he termed the Biologically Oriented Preparation Technique (BOPT). The author defines this technique as a simplified prosthetic protocol that employs vertical “feather edge” preparation in the initial phase of treatment, followed by the immediate placement of a provisional restoration. This provisional restoration plays a fundamental role in the stabilization of the gingival tissues (2).

The provisional crown, placed immediately after the preparation, facilitates the healing of the surrounding tissues in accordance with its shape. Loi has demonstrated, through extensive research, that the gingiva adapts to the shape of both the provisional crown and the final restoration over time. This is accomplished by reshaping the emergence profiles and adapting the gingival scallops obtained by the new prosthetic shape (2).

Upon completion of the temporary crown and subsequent fabrication of the final restoration, two potential issues may arise:

• The analog or digital impression may not accurately reflect the shape of the gingiva obtained with the temporary crown due to gingival collapse upon crown removal, even in the presence of retraction cords.

• The desired gingival shape may not have been achieved with the temporary crown.

At this juncture, the laboratory technician assumes a pivotal role in fabricating the final crown according to the clinician’s specifications. These specifications include the crown shape, emergence profile, gingival zenith, etc. As previously stated, the gingiva will adapt to the new prosthetic shape (2).

The bisque try-in offers the opportunity to ascertain whether the information provided to the laboratory was sufficient to fabricate a crown that meets the clinician’s expectations. Nevertheless, it is possible that, at the time of delivery of the final restoration, the gingival contour may not exhibit the optimal shape or the correct zenith position. This is the point at which minor modifications to the morphology of the new restoration can be made in the clinic, with the aim of facilitating adaptation of the tissues to the shape of the final restoration.

The objective of this article is to describe the stabilization of gingival tissues following twelve weeks of clinical observation. During this period, the provisional crown is recontoured twice in the apical-coronal direction with a four-week interval. This approach allows for the acquisition of sufficient gingival tissue in the horizontal direction at the point of the vestibular emergence profile, which will then stabilize once more following a slight recontouring of the final restoration, which will be performed in the clinic.

## Case Report

**Figure 1:**
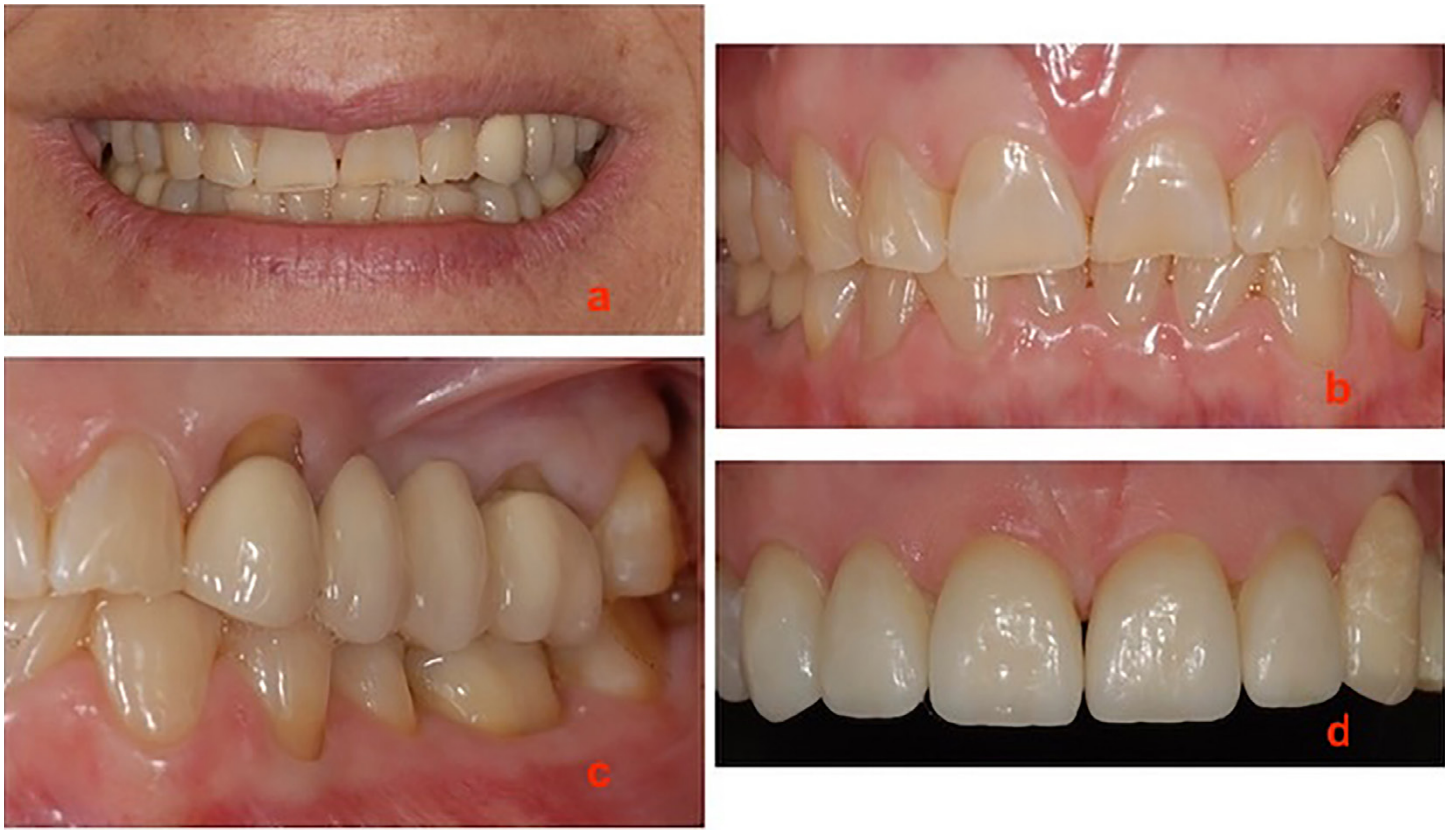
a-c) Initial clinical photos showing the apical displacements of the gingival tissue. d) Temporary bridge.

A 60-year-old female patient presented to the clinic with generalized wear on all her teeth, which was attributed to bruxism. Upon intraoral examination, it was observed that the patient had a metal-ceramic bridge on teeth 23 to 26, with teeth 24 and 25 absent. Furthermore, a noTable gingival displacement was observed at the cervical region of tooth 23. The proposed treatment plan for the patient includes a complete rehabilitation with minimally invasive restorations in both the anterior and posterior sectors. In the anterior sector, ceramic veneers will be utilized, while in the posterior sector, composite overlay restorations will be employed to restore the vertical dimension lost as a result of bruxism. Additionally, the metal ceramic bridge will be replaced with a zirconia-ceramic bridge. This will entail attempting to migrate the gingival tissues in an apical-coronal direction using the BOPT technique, after which they will be stabilized, obviating the need for a periodontal graft. (Fig. 1a-c).

-Clinical sequence

Following the removal of the old bridge, the abutment teeth are prepared with a vertical preparation. A temporary bridge is then placed and kept in place for four weeks, in accordance with the steps described in the BOPT technique (2). In this case, the temporary bridge is provided with a slightly more accentuated emergence profile to achieve a greater amount of gingival tissues horizontally (Fig. 1d). After this time, it is observed that a considerable increase in the gingival tissues horizontally has been achieved. As the soft tissues adapt to the shape of the prosthesis and the intention of the treatment is to modify the gingival parabola in an apical-coronal direction, it is necessary to lower the contour of the emergence profile of the provisional prosthesis by 1mm every four weeks.

**Figure 2:**
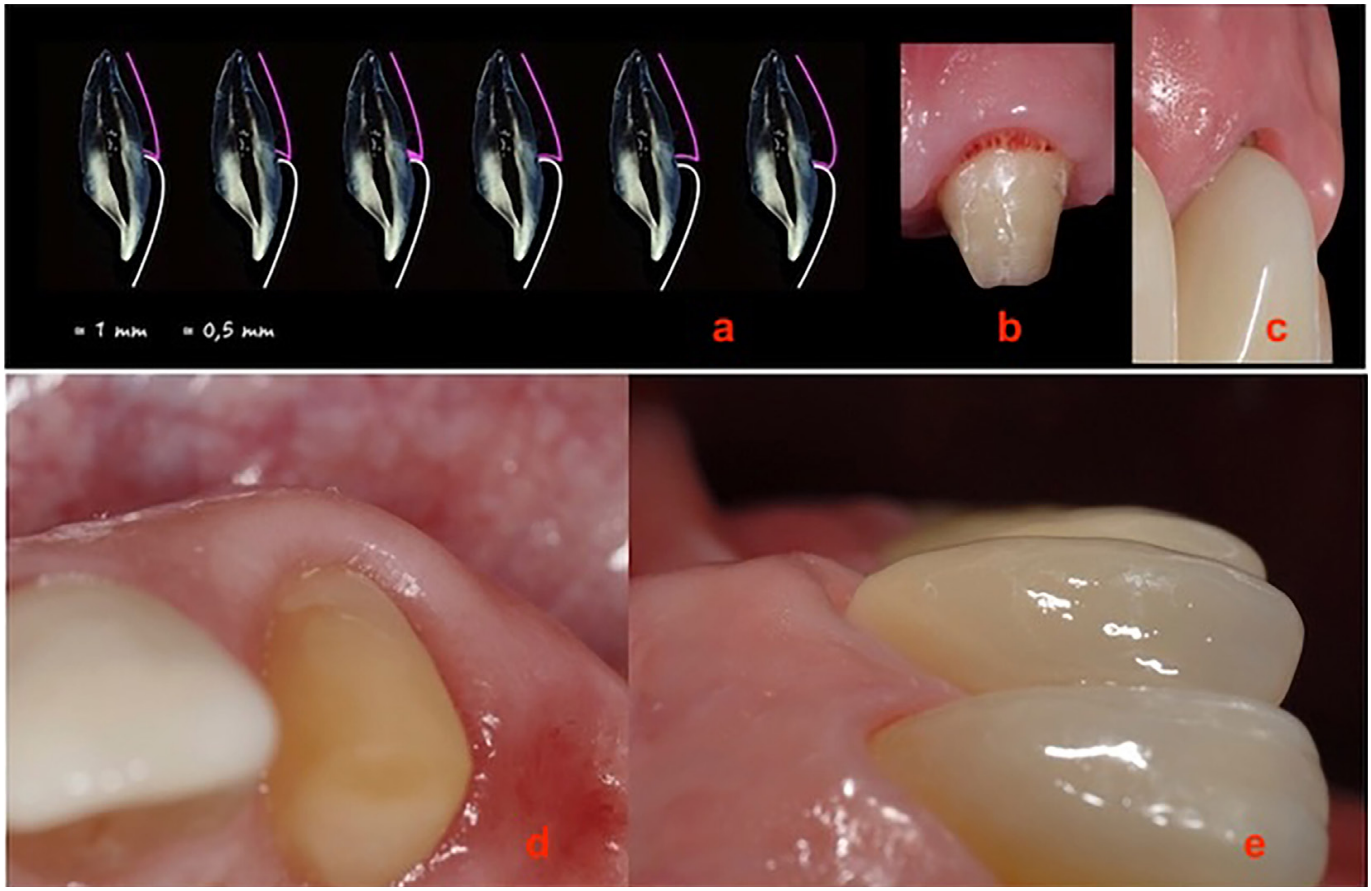
a) Scheme of the horizontal-vertical platform; b) Neo angiogenesis; c) Adaptation of the gingival parabola to the new emergence profile, reduction of gingival thickness in the horizontal direction; d) Amount of tissue that was obtained in a horizontal direction; e) The contour of the final prosthesis was lowered in an apical-coronal direction.

The extent of tissue adaptation to the novel emergence profile is directly proportional to the quantity of tissue that has been successfully obtained horizontally (Fig. 2a).

The image (Fig. 2b,c) illustrates a good adaptation of the gingival contour to the prosthetic morphology determined by the temporary bridge. This adaptation was achieved by lowering the bridge contour by 1mm every 4 weeks, with the objective of leveling the gingival parabola of tooth 23 to that of tooth 13. Following the recontouring of the provisional bridge twice, it was determined that the position of the gingival margin at 23 was comparable to that of the contralateral side, and a final impression was taken for the fabrication of a zirconia-ceramic bridge from 23 to 26 by a laboratory technician. Upon receipt of the completed prosthesis, a slight asymmetry was observed between teeth 23 and 13. This prompted the decision to recontour the emergence profile of the final prosthesis in the position of tooth 23, due to the amount of available gingival tissue in the horizontal plane. Therefore, the contour of the final prosthesis was lowered in an apical-coronal direction (Fig. 2d). This is done on a horizontal-vertical platform, with a reduction of approximately 1mm of the zirconia-ceramic, using a 120-micron truncated conical bur in a high-speed handpiece and abundant water. Subsequently, the ceramic is manually polished using a handpiece and specific cups and discs designed for this purpose. Finally, the bridge is cemented with temporary cement.

In this context, it is of paramount importance that a thorough manual polishing of the ceramic be conducted following the high-speed handpiece recontouring. This should be done using specific polishing pastes and cups.

**Figure 3:**
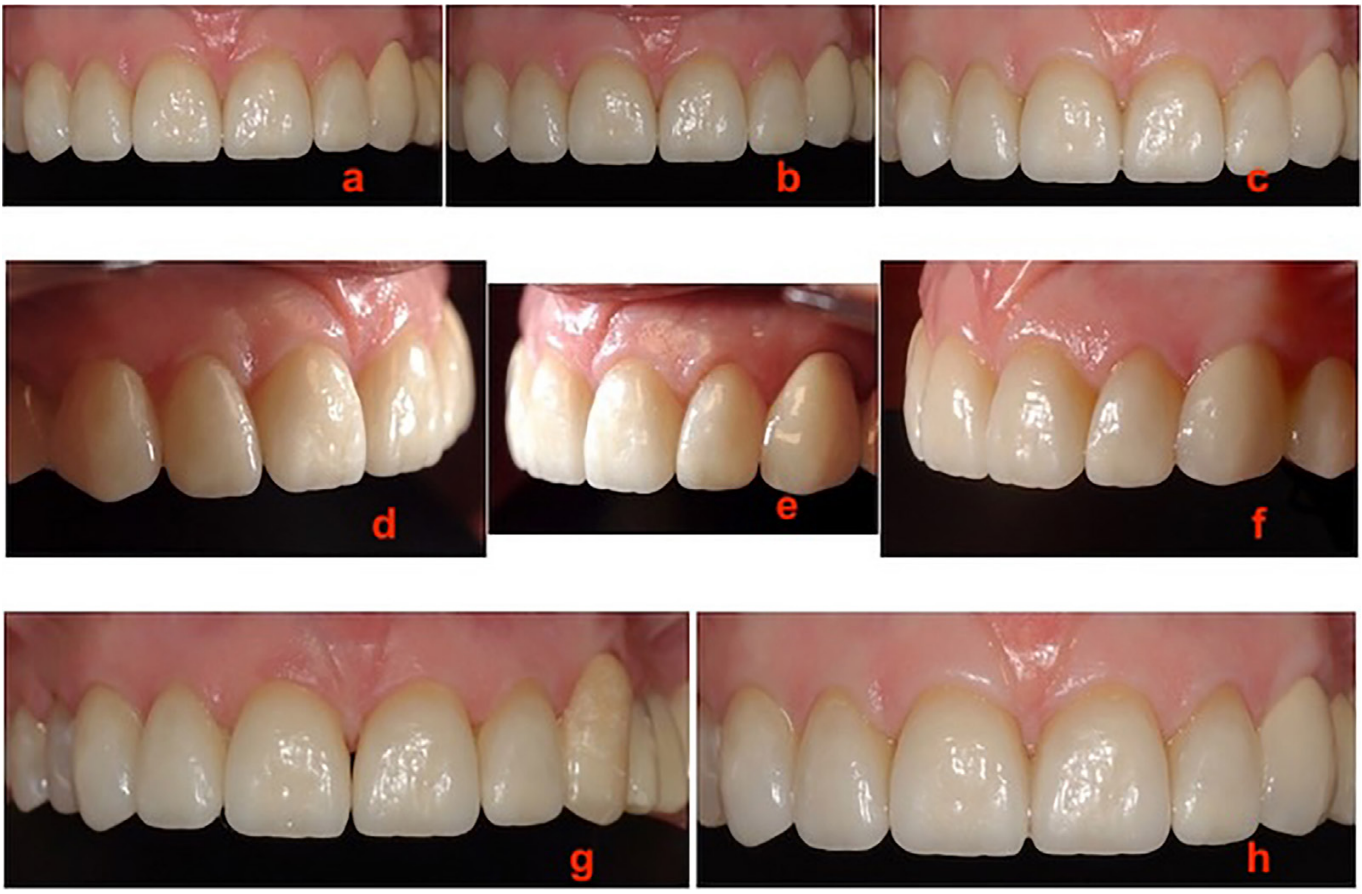
a) The bridge before ceramic recontouring; b) Juncture of ceramic recontouring; c) At 12 months, the gingival parabola adapts the to the new prosthetic shape, because there is adequate tissue availability in the horizontal direction; d and f) A symmetry is observed at the level of the gingival parabolas of 13 and 23; e) Juncture at which ceramic recontouring takes place; g) The first temporary bridge; h) Situation at 12 months.

At one week, the patient was examined and a slight displacement of the gingival tissues in an apical-coronal direction was already evident, adapting to the new emergence profile of the final restoration. After three months, an optimal aesthetic situation was observed in which the gingival parabola of tooth 23 was aligned with its contralateral tooth 13. This corroborates our hypothesis that the gingival parabola adapts the to the new prosthetic shape, if there is adequate tissue availability in the horizontal direction (Fig. 3).

## Discussion

In horizontal “knife-edge” dental preparations, i.e. with a finishing line, where the final restoration is cemented at that level, it has been observed over time that apical displacements of the gingival tissues may occur. This can be attributed to various factors, including iatrogenic damage during dental preparation, failure of restoration adjustment at this level, traumatic factors such as occlusion, an incorrect and/or aggressive brushing technique, or it can even be related to the periodontal phenotype of the patient (9-12).

In contrast, the BOPT technique described by Loi, which involves the creation of vertical “feather-edge” preparations, does not have a defined “finishing line”. Instead, it has a “finishing area”. The laboratory technician is responsible for determining the starting line of the restoration, based on the information provided by the gingival tissues (2). Restorations with vertical preparations have been demonstrated to offer superior periodontal health compared to restorations with horizontal preparations (4).

The gingitage performed during vertical preparation and the use of a provisional restoration are essential for thickening and stabilizing the gingival tissues (13,14). In the original BOPT technique, Loi recommends recontouring the temporary restoration to correct the gingival margin coronally or apically (2). Given that soft tissues continue to remodel and adapt to the shape of the final crown over time (15), and the availability of sufficient tissue in the horizontal direction, it was decided to directly recontour the emergence profile of the final crown.

## Conclusions

According to the authors’ experience, the amount of gingival adaptation is not quantifiable in a numerical sense; rather, it is directly proportional to the amount of tissue that can be obtained with the new emergence profile of the temporary crown. The outcome is contingent upon the operator and there is no fixed quantity that can be achieved in every instance. In essence, there is no fixed numerical value that can be relied upon to lower the gingival parabola in the apical-coronal direction through the adaptation of tissues to the new shape of the temporary crown emergence profile.

The amount of tissue that will gradually adapt, through the adjustments of the temporary prosthesis, will decrease until it stabilizes (reducing horizontally) with an ideal shape that will depend on the anatomy of the complex formed by the prosthetic element, the supporting tissue, and the new prosthetic emergence profile.

Once the final prosthesis has been cemented, the gingival tissues will continue to remodel and adapt to the new prosthetic shapes. The embrasure spaces will subsequently close, and the gingival margin will thicken and assume a “gull-wing” silhouette.

Further clinical studies with patients are necessary to confirm and contrast these results.

## Data Availability

The datasets used and/or analyzed during the current study are available from the corresponding author.
